# A Girl’s Toilet Articles

**Published:** 1889-07

**Authors:** 


### A GIRL'S TOILET ARTICLES.

A sensible girl will not keep a lot of cosmetics and drugs on her toilet table, but there are a few articles she should always have in a convenient place. She should have an array of glass stopped bottles containing alcohol, alum, camphor, borax, ammonia and glycerine or vaseline. A little camphor and water may be used as a wash for the mouth and throat if the breath is not sweet. Powdered alum applied to a fever sore will prevent it from becoming very unsightly and noticeable. Insect stings or eruptions on the skin are removed by alcohol. A few grains of alum in tepid water will relieve people whose hands perspire very freely, rendering them unpleasantly moist. A few drops of sulphuric acid in the water are also beneficial for this purpose, and are also desirable for those whose feet perspire freely. We should always recommend care in the use of scented soap; in many cases the perfume is simply a disguise for poor quality. A good glycerine or honey soap is always preferable. Of course, one may rely on scented soap from a high-class manufacturer, but it costs more than it is worth. In addition to the soap for bathing, white castile should be kept for washing the hair. Occasionally, a little borax or ammonia may be used for this purpose, but it is usually too harsh in its effects.

